# Physical health and cognitive ability factors in predicting retirement adjustment based on machine learning approach: results from the China Health and Retirement Longitudinal Study

**DOI:** 10.3389/fpsyg.2025.1601723

**Published:** 2025-08-20

**Authors:** Chenhong Cui, Bolin Zhou, Lin Gao, Dahua Wang, Angela C. Rowe, Bruna S. Nascimento, Hao Zhang, Xiancai Cao

**Affiliations:** ^1^Faculty of Psychology, Tianjin Normal University, Tianjin, China; ^2^School of Management, Tianjin Normal University, Tianjin, China; ^3^Institute of Developmental Psychology, Beijing Normal University, Beijing, China; ^4^School of Psychological Science, University of Bristol, Bristol, United Kingdom; ^5^Key Research Base of Humanities and Social Sciences of the Ministry of Education, Academy of Psychology and Behavior, Tianjin Normal University, Tianjin, China; ^6^Tianjin Social Science Laboratory of Students' Mental Development and Learning, Tianjin, China

**Keywords:** retirement adjustment, physical health, cognitive ability, depression, life satisfaction, machine learning

## Abstract

**Introduction:**

Retirement is one of the most significant status changes in an individual's later life. Physical health and cognitive ability are key predictors of retirement adjustment. However, studies have yet to investigate the role of different physical health and cognitive ability indicators simultaneously, and their non-linear association in relation to retirement adjustment.

**Methods:**

This study used machine learning methods to explore the predictive role of both physical and cognitive ability variables in retirement adjustment. Using longitudinal data from the China Health and Retirement Longitudinal Study (CHARLS) database, a total of 1,314 participants met the retirement criteria, and the increase in life satisfaction and decrease in depression scores were extracted as the indicators of successful retirement adjustment. Various physical health and cognitive ability-related variables measured before retirement, alongside key demographic and lifestyle variables, were used as predictive variables to predict retirement adjustment 2 or 3 years later. Random forest (RF) and XGBoost classification models were used as predictors, and SHAP (SHapley Additive explanation) value analysis was used to explain the model prediction results.

**Results:**

The results indicated that the accuracy of the RF and XGBoost models outperformed regularized logistic regression. Self-rated hearing, income, attention and calculation ability, self-rated health, and time orientation ability were identified as the most influential predictors of retirement adjustment. Self-rated memory and sleep duration exhibited a non-linear relationship with retirement adjustment.

**Discussion:**

The present research extends current understanding of factors that promote adjustment to retirement and provides essential insights for preventing poor adjustment and intervening in retirement adjustment.

## 1 Introduction

Retirement is one of the most important status changes in an individual's later life (Stephan et al., 2008), which affects an individual's life satisfaction (Hansson et al., 2018), depressive symptoms (Segel-Karpas et al., 2018), and wellbeing (Wang, 2007). As such, the issue of how individuals adjust to retirement is a focus of interest for researchers (Wang and Shultz, 2010). Because research on retirement adjustment can directly inform how to improve quality of life after retirement (Wang et al., 2011), understanding the predictors of retirement adjustment is critical for helping individuals transition successfully.

Previous research has found that physical health variables, such as self-rated health (Earl et al., 2015; van Solinge and Henkens, 2008) and number of illnesses (Zhan et al., 2023), as well as cognitive ability variables, such as self-rated cognitive ability (Hansson et al., 2019, 2020), general objective cognitive ability (Zhan et al., 2023), and abstract reasoning (Denier et al., 2017) are powerful and important factors predicting retirement adjustment. However, most studies of predictors of retirement adjustment to date have adopted a linear perspective, that is, they have used traditional multivariate analyses, such as linear regression and factor analysis, which assume that relationships between variables are linear. This approach works well for simple datasets but faces challenges in more complex contexts. Specifically, traditional multivariate analyses perform poorly when dealing with non-linear models, where the relationships between variables are more intricate and cannot be captured by straight lines. Furthermore, these approaches struggle to handle large-scale and high-dimensional data, which refers to datasets with many features or variables relative to the number of observations (Cooray et al., 2021). Previous linear models have shown limited variance explanation rates in predicting retirement adjustment (Kaveh et al., 2022), which is suboptimal for accurate prediction and effective intervention. Furthermore, existing studies have yet to simultaneously examine the effects of specific physical health and cognitive ability indicators, which have been previously identified as critical for predicting retirement adjustment. Importantly, the potential non-linear relationship between physical health and cognitive ability, and retirement adjustment has been overlooked in previous literature.

To address this gap, this study investigates the predictive roles of a range of specific physical health and cognitive ability variables, along with other relevant lifestyle and demographic factors in retirement adjustment simultaneously. It also aimed to capture the non-linear relationships between these variables and thereby enhance predictive accuracy to support the detection of early warning signs of retirement adjustment problems and intervention strategies. By employing machine learning algorithms and using longitudinal data, our study extends previous findings and methodology, advancing current understanding of the contributions of physical health and cognitive ability to retirement adjustment.

## 2 Literature review

### 2.1 Retirement adjustment theory

Retirement is a significant transition in a person's life and is often considered a sign of entering old age (Bauger and Bongaardt, 2016). It has been conceptualized as a decision-making process, an adjustment process, or a career development stage (Wang and Shultz, 2010; Wang and Shi, 2014). The issue of how individuals adjust to retirement has been a focus of interest for researchers since the 1930s (Linton, 1936; Wang and Shultz, 2010). The retirement adjustment process incorporates both the retirement transition (i.e., from employment to retirement) and postretirement trajectory (i.e., postretirement development in life) (Wang, 2007; Wang et al., 2011). In particular, this conceptualization emphasizes that it is not the decision to retire, but rather the characteristics of the retirement transition process embedded in this decision, that are of most importance (van Solinge and Henkens, 2008). Thereby highlighting the importance of preretirement predictors. Good retirement adjustment is characterized by high life satisfaction, low depressive symptoms (Chao et al., 2022), and high wellbeing, and can promote successful aging (Bauger and Bongaardt, 2016).

Several theories have been put forward to explain retirement adjustment. Role theory is an early model that explains retirement adjustment in terms of the importance of job roles (Linton, 1936), and the impact that change or loss of these roles will have on retirement motivation, values, and intentions (Ashforth, 2001; Ashforth et al., 2008). More recent approaches have proposed that retirement adjustment is a longitudinal and developmental process (La Rue et al., 2022; Wang et al., 2011). Among them, the life course perspective recognizes that the adjustment process to retirement is influenced by individual characteristics (Kim and Moen, 2002), personal past experiences (Orel et al., 2004), and accumulated knowledge and the social ties of work (van Solinge and Henkens, 2008). Wang et al. (2011) proposed a resource-based dynamic perspective, which posits that retirement adjustment is a longitudinal process and retirees' levels of adjustment may fluctuate with change in individual resources. Among these resources, physical (e.g., self-rated health, illness), cognitive (e.g., memory, attention), financial (e.g., retirees' financial status, unemployment before retirement), and social resources (e.g., retirees' marital status, marital quality) are the most important resources to predict retirement adjustment (Leung and Earl, 2012; Wang and Shi, 2014). Thus, in contrast to the theories reviewed earlier, which tend to focus narrowly on a single aspect of retirement adaptation—such as emphasizing either its dynamic nature or the influencing factors without accounting for their interplay over time—the resource-based dynamic theoretical framework provides a holistic perspective. It not only integrates various layers of influencing factors but also takes into account the dynamic changes inherent in the process of retirement adjustment.

Similarly, the retirement transition and adjustment framework (RTAF) emphasizes the impact of individual differences and dynamic interplay between resources on retirement adjustment (Hesketh et al., 2011, 2015). It emphasizes the fit between individuals and their environments, incorporating structural (abilities, needs) and dynamic (adaptability, proactive/reactive behaviors) components over time. The framework accounts for diverse adjustment trajectories, influenced by individual differences, family, societal, and policy-level factors. Among these factors, The RTAF posits that individuals' physical and cognitive abilities are the necessary capacities required for adapting to the environment afterretirement (Hesketh et al., 2011, 2015). In summarizing the above theories, we conclude that individuals' physical and cognitive abilities before retirement are very important. It is worth noting that relevant theories also highlight the necessity to draw on pre-retirement factors when examining adjustment outcomes in retirement. The Resource-based Theory highlights that the quantity and quality of tangible and intangible resources available before retirement are critical predictors of successful adjustment post-retirement (Wang and Shultz, 2010). Life Course Theory emphasizes that individuals' prior experiences, accumulated resources, and health status throughout their lives shape their capacity to adapt after retirement (Elder Jr, 1995). Moreover, Transition Theory frames retirement as a major life transition wherein the degree of preparation, coping mechanisms, and available support systems prior to the transition significantly influence the adaptation outcomes (Schlossberg, 1981). Together, these suggest that retirement adjustment cannot be fully understood without considering the pre-retirement context and resources, as they provide the foundation upon which post-retirement adaptation is built. Since retirement transition involves a major shift in identity, it is a critical phase of adaptation (Reitzes and Mutran, 2004). What's more, the current study emphasizes the importance of personal resources before retirement, making it more meaningful to identify predictive factors that can guide individuals in preparing for retirement. The current study primarily focused on retirement adjustment during the transition phase. Therefore, our study is based on longitudinal data to explore the predicting role of physical health and cognitive ability before retirement on retirement adjustment during the retirement transition phase.

### 2.2 Effects of physical health and cognitive ability on retirement adjustment

Several researchers have summarized and reviewed the predictors of retirement adjustment (Reitzes and Mutran, 2004; Barbosa et al., 2016; Yeung and Zhou, 2017; Amorim and França, 2019; La Rue et al., 2022). For example, a systematic review by Barbosa et al. (2016) found that physical health had the highest positive effect on retirement adjustment relative to other resources such as finances, leisure, and social integration. Similarly, a meta-analysis by La Rue et al. (2022) found a strong correlation between physical health (including self-report or objective ratings), cognitive ability (e.g., memory impairment), and retirement adjustment.

Indeed, multiple studies have demonstrated the impact of physical health on retirement adjustment. Specifically, subjective perceived health has been found to be positively associated with retirement adjustment (Earl et al., 2015), retirement satisfaction (van Solinge and Henkens, 2008), and post-retirement wellbeing (Gall et al., 1997; Kim and Moen, 2002). From an objective health perspective, Zhan et al. (2023) found the number of physical diseases before retirement affected changes in life satisfaction after retirement. In conclusion, most previous studies used self-rated health, or number of diseases as physical health indicators. Few studies have examined the direct effects of different specific physical health indicators on retirement adjustment.

However, research has examined the impact of these specific indicators on early retirement or retirement life. For example, Kang et al. (2015) found that participants who suffered from hypertension, diabetes, malignancy, heart disease, stroke, or arthritis had a significantly higher risk of health-related early retirement. In addition, persistent shoulder pain was a predictor of voluntary early retirement (Jensen et al., 2016). Tooth loss was significantly associated with multiple chronic diseases (Hag Mohamed and Sabbah, 2023) and self-rated health (Barboza-Solís et al., 2019), which affect retirement life. In addition to chronic diseases, health-related lifestyles were also found to be associated with retirement adjustment, such as alcohol use (Perreira and Sloan, 2001) and smoking (Henkens et al., 2008). The above studies have shown that different physical health variables impact retirement life. However, the relative importance of these detailed physical health indicators for retirement life, particularly successful retirement adjustment, has not yet been studied.

Cognitive ability also plays a vital role in retirement adjustment. Previous research found that self-rated cognitive ability positively correlated with life satisfaction in retirement (Hansson et al., 2020) and can even compensate the negative impact of poor health on life satisfaction after retirement (Hansson et al., 2019). Additionally, general objective cognitive ability among older adults could positively predict the postretirement change trajectory of life satisfaction through retirement transition experience (Zhan et al., 2023), such that individuals with higher general objective cognitive ability tend to have a better retirement transition experience, leading to more positive changes in life satisfaction. As is the case with physical health, when it comes to cognitive ability, most studies have used self-rated and general cognitive ability rather than specific objective cognitive indicators to examine cognitive impacts on retirement adjustment.

In summary, previous research has focused on self-rated or general physical health and cognitive ability and has found that physical health and cognitive ability strongly and positively relate to retirement adjustment. However, limited studies have focused on the impact of specific physical health and cognitive ability domains on retirement adjustment simultaneously or compared their importance in predicting retirement adjustment.

In addition, most extant studies used a linear perspective to explore the relationship between physical health and cognitive ability and retirement adjustment. However, Schilling and Wahl (2006) pointed out that emotional wellbeing implies non-linear intraindividual trajectory changes that are substantially related to disease duration. Liu et al. (2024) found that patients with a persistent combination of pain, depression, and frailty first exhibited fan improvement but then worsened over time. Therefore, the impact of health variables on retirement adjustment may be non-linear and varied, as argued above. Additionally, the linear model has had poor performance in explaining retirement adjustment, and its variance explanation rate is limited. For example, Leung and Earl (2012) found that retirement resources accounted for 22% of the total variance in retirement adjustment. Relevant theories also indicate that predictors may exhibit non-linear relationships with retirement adjustment. The Resource-based Dynamic Model (Wang and Shultz, 2010) suggests that individuals draw upon various resources—such as financial, social, and health-related—which may show diminishing returns or threshold effects in their impact on adjustment. Similarly, Socioemotional Selectivity Theory proposes that aging individuals shift their goals and emotional regulation strategies over time (Carstensen, 1995), meaning that the same predictor may have different effects as individuals' attitudes and social goals change. The Selective Optimization with Compensation model (Baltes and Baltes, 1990) further explains how individuals adapt through flexible strategies that are sensitive to context and personal capacity, which may create curvilinear or tipping-point effects. These frameworks indicate predictors may exhibit non-linear effects on retirement adjustment. However, the non-linear relationships between physical health, cognitive ability, and post-retirement adjustment warrants exploration.

### 2.3 Current study

The above literature review reveals that previous studies lack an examination of specific physical health and cognitive ability indicators that predict retirement adjustment and the comparison of these predictors. Traditional linear regression's performance in predicting relationships between them has been relatively poor and has ignored the non-linear effect of predictors such as chronic disease. It is necessary to further explore the relationship between discrete physical health and cognitive ability indicators and retirement adjustment.

Traditional multivariate models have statistical limitations, such as poor performance in complex and non-linear relationships. They may also face computational challenges in dealing with large-scale and high-dimensional data (Cooray et al., 2021). When there are non-linear relationships, interactions, or high-dimensional features in the data, the predictive performance of machine learning models is usually better than that of traditional linear regression (Bzdok et al., 2017). Compared to traditional statistical methods, machine learning-based models go beyond the regression modeling framework, excelling at capturing non-linear relationships. Further, machine learning models can make accurate predictions while considering a more comprehensive range of factors and can quickly identify the most critical predictors in high-dimensional feature sets without making strict assumptions about the predictor and the outcome (Breiman, 2001; Bzdok et al., 2017). Despite these advantages, a significant challenge is that machine learning models are considered a “black box” (Pargent et al., 2023); that is, the model makes it difficult to elucidate how predictions are made and thus may not be suitable for obtaining actionable explanations (Bi et al., 2019). Fortunately, with the development of more interpretable methods, such as the SHapley Additive exPlanations (SHAP; Lundberg and Lee, 2017), researchers can better understand the impact of these features on model predictions (Pargent et al., 2023). Therefore, machine learning methods are well suited to using multiple predictive indicators. Previous studies have used the method of machine learning to conduct exploratory research on retirement decision-making, and the results emphasized health as a factor in retirement decisions; poor health was associated with early retirement (Garibay et al., 2022). Retirement adjustment differs from retirement decision-making, which are two different stages of retirement. We simultaneously used multiple physical health and cognitive ability indicators to predict retirement adjustment status by machine learning methods.

To investigate the relationship between physical health and cognitive ability indicators to predict retirement adjustment, the current study used the China Health and Retirement Longitudinal Study (CHARLS) database, a longitudinal tracking data, to understand the relationship between variables better. The database is designed to collect the health status of middle-aged and elderly people in China, and it contains a relatively wide range of physical health and cognitive ability variables. In addition to the traditional physical health and cognitive variables, the current study incorporates health-related lifestyle factors and demographic variables to predict retirement adjustment in the database. Previous research has highlighted that health-related lifestyle factors, such as alcohol use (Perreira and Sloan, 2001) and smoking (Henkens et al., 2008), as well as demographic variables, particularly income and education (Zhan et al., 2023), are strongly associated with retirement adjustment. The random forest (RF) classification algorithm and extreme gradient boosting (XGBoost) classification algorithm were used for retirement adjustment prediction, and the logistic regression algorithm was used as a reference model. The main aim of our study was to investigate the relationship between physical health and cognitive ability and retirement adjustment using a non-linear perspective through machine learning modeling.

## 3 Methods

### 3.1 Study design and participants

**Figure 1:**
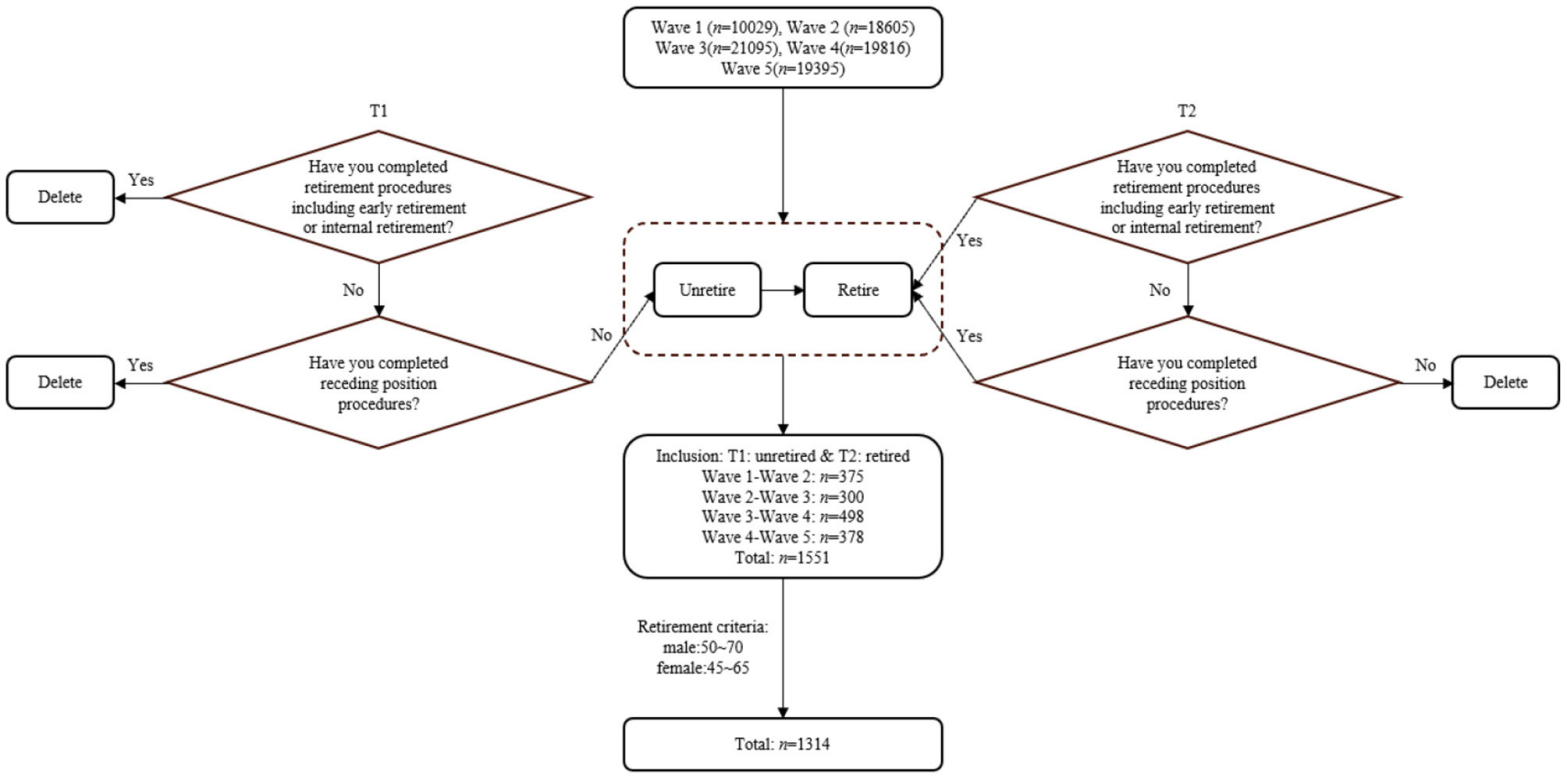
Participants selection process. *Receding procedure refers to the case where the employee is not eligible to retire in terms of age or length of work years, but has lost the ability to work due to illness or disabilities and hence needs to withdraw from his/her previous position. According to the law the employee shall receive a certain amount of compensation in this scenario.

The study is based on longitudinal data from the China Health and Retirement Longitudinal Study (CHARLS) database (Zhao et al., 2014). The database aimed to collect health status information on middle-aged and elderly individuals (primarily aged 45 and above) across 28 provinces in China, incorporating five waves of data gathered in 2011, 2013, 2015, 2018, and 2020. Considering that retirement adjustment is a gradual process, participants who met the following criteria were included: (1) not retired at wave n, and (2) retired at the subsequent wave *n* + 1. Retirement status was determined based on two questions in each wave of data. Meeting one of these conditions is retirement; failing to meet both conditions is not retirement. The questions are: (1) Have you completed retirement procedures (including early retirement) or internal retirement? (2) Have you completed receding procedures?^*^ A total of 1,551 participants were selected. The retirement system of China stipulates that the normal retirement age for males is 60 years old, and the age for early retirement due to special reasons is 55 or 50 years old. For females, the retirement age is 55 or 50, and the age for early retirement due to special reasons is 45. Furthermore, with the aging population, delayed retirement policies have been introduced in some cases. Therefore, according to the statutory retirement age and the practice of most scholars (Feng and Han, 2017; Zheng and Wang, 2020), the age range of our screening was 50–70 years old for males and 45–65 years old for females (Figure 1 for participants selection process). According to the age criteria, 1,314 participants were selected at last. In the selected sample, the average age of participants was 56.48 (SD = 5.63). The majority were men (53.88%), lived in an urban environment (72.53%), were literate (66.11%), and were married (93.46%).

### 3.2 Outcome variables and input variables

#### 3.2.1 Outcome variables

**Figure 2:**
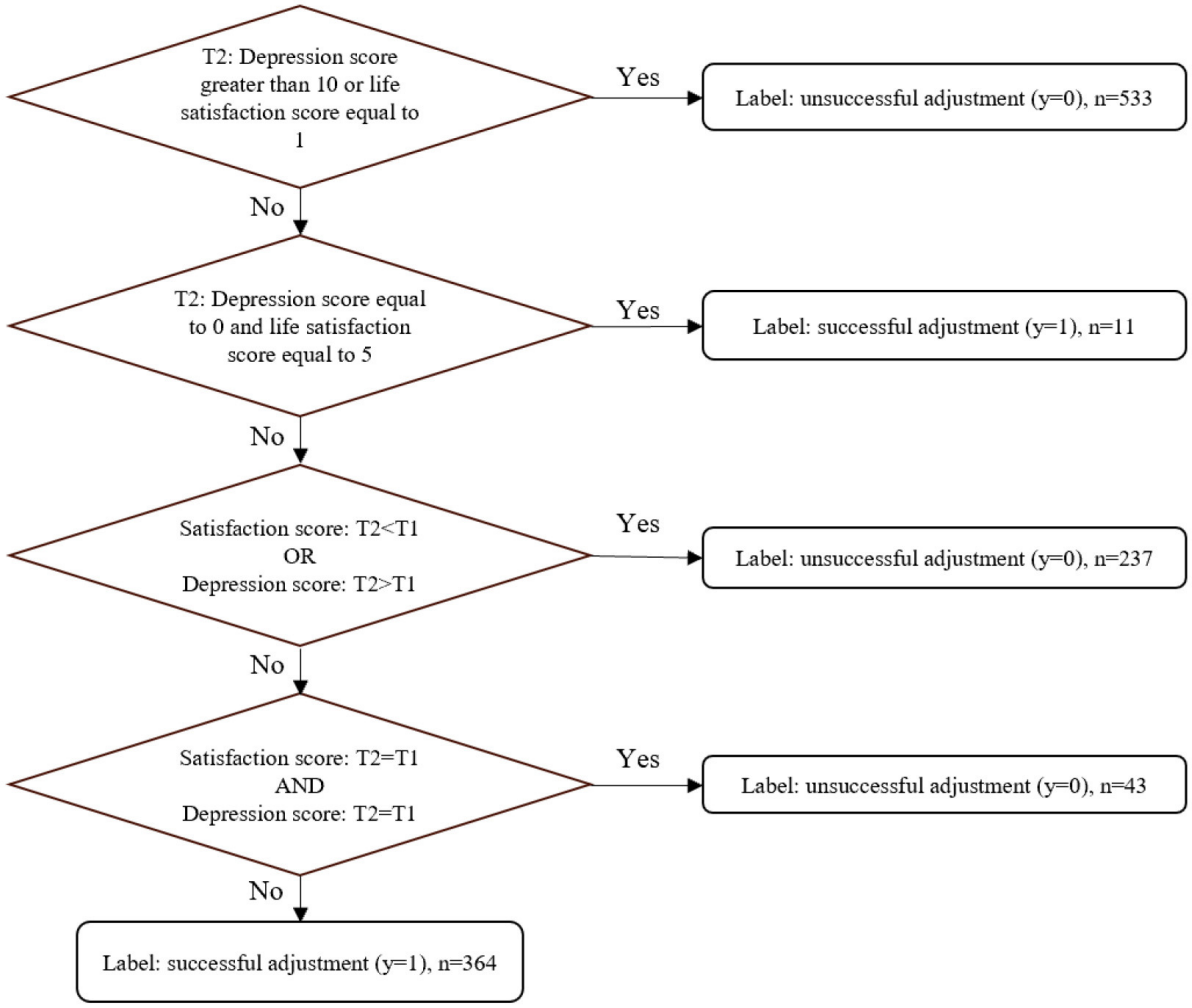
Labeling process of outcome variables.

This study defined the outcome variable as whether retirement adjustment was successful. A large body of literature has examined the extent to which individuals feel psychologically comfortable and adapt to the changed retirement environment (Wang et al., 2011). The indicators of psychological comfort include happiness (Calvo et al., 2009; Ju et al., 2017), wellbeing (Wang, 2007; Barrett and Kecmanovic, 2013), retirement satisfaction (Stephan et al., 2008; Price and Balaswamy, 2009), life satisfaction (Hansson et al., 2018; Dingemans and Henkens, 2015), and mental health and depression (Segel-Karpas et al., 2018), among others. Chao et al. (2022) noted that high life satisfaction and low depressive symptoms represented good retirement adjustment. Therefore, our study used the changes in satisfaction and depression as the indicator of retirement adjustment status. The satisfaction score was collected based on the questions in each wave of data: Please think about your life as a whole, how satisfied are you with it? 1= not at all satisfied, 5= completely satisfied. The depression score was collected using the Center for Epidemiologic Studies Depression Scale (CSE-D; Andresen et al., 1994). The scale includes two positive items and eight negative items. Each question had four options: “Rarely or none of the time,” assigned 0; “Some or a little of the time,” assigned 1; “Occasionally or a moderate amount of the time,” assigned 2; “Most or all of the time,” assigned 3. A total score exceeding 10 after reversing the scores of positive items represents depression. First, at T2, the depression or satisfaction scores were in a poor state (depression score >10 or life satisfaction score = 1), they were labeled unsuccessful retirement adjustments (*y* = 0). When depression and satisfaction scores were in the best state simultaneously (depression score = 0 and life satisfaction score = 5), the state was labeled as successful retirement adjustment (*y* = 1). Then, the state in which satisfaction scores at T2 were < T1 or the depression score at T2 was >T1 was labeled as unsuccessful retirement adjustment (*y* = 0). Finally, the state in which both satisfaction and depression scores remained unchanged was labeled as unsuccessful retirement adjustments (*y* = 0). Otherwise, it was considered a successful adjustment (*y* = 1). The specific labeling process of outcome variables is shown in Figure 2.

#### 3.2.2 Input variables

Based on the dynamic resource retirement theory, literature reviews, and data availability in the current database, 37 input variables were selected. These variables were categorized as follows: (1) Demographic factors: age, gender, area type, education, marital status, and income. (2) Objective physical health factors: including 12 chronic diseases, five disability symptoms, pain, tooth loss, and falls. (3) Subjective physical health factor: self-rated health, self-rated distance vision, self-rated close vision, and self-rated hearing. (4) Health-related lifestyle factors: smoking, drinking, and sleep duration. (5) Cognitive ability factors: time orientation ability, attention and calculation ability, immediate memory ability, and self-rated memory. These factors were extracted from the different cognitive abilities measured by Mini-Mental State Examination (MMSE). Please refer to Appendix B for details of all measures.

### 3.3 Data analysis

#### 3.3.1 Oversampling

Data analysis was performed using R 4.3.3 and Python 3.9.

The “mice” package (Buuren and Groothuis-Oudshoorn, 2011) in R 4.3.3 was utilized for multivariate imputations by chained equations (MICE) to address missing data. Missing values (the missing value of income is 18%, the missing value of the remaining variables are < 9%) in the dataset were imputed using a method based on the random forest algorithm, which has become a popular and effective approach for analyzing datasets with missing values (Little and Rubin, 2002). Random forest-based MICE can deal with various types of large-scale data with a high number of features and complex non-linear relationships, it is more efficient, produce narrower confidence intervals and predict missing values more accurately than standard implementation of MICE (Shah et al., 2014). According to the complete dataset, the incidence of successful retirement adjustment was 30.29% (minority class) as opposed to 69.71% (majority class) for unsuccessful adjustment. This inconsistency between categories can lead to a poor-performing machine learning model due to the unequal representation of the two categories of participants (Wiemken and Kelley, 2020). Oversampling of the minority class is a commonly used procedure with imbalanced data (Cooray et al., 2021). Therefore, random oversampling of the minority category was performed to equalize the sample size between adjusted and non-adjusted individuals.

#### 3.3.2 Feature selection

The random forest permutation importance selection algorithm was used to further reduce the dimensionality of the data and select only the most relevant variables for the final model (Ramosaj and Pauly, 2023). We selected the top 18 features above 0.001 (see Appendix A). The final selected predictors included three demographic factors (age, gender, and income), four objective physical health factors (stomach, dyslipidemia, pain, and fall), four subjective physical health factors (self-rated health, self-rated distance vision, self-rated close vision, and self-rated hearing), three health-related lifestyle factors (smoking, drinking, and sleep duration) and four cognitive ability factors (self-rated memory, time orientation ability, attention and calculation ability, and immediate memory ability) and more detailed descriptions of input variables and assignments of variables are presented in Appendix B. The physical health and cognitive ability-related and demographic variables at wave *n* were used as predictive variables to predict the retirement adjustment at subsequent wave *n* + 1.

#### 3.3.3 Predictive model development and evaluation

Random forest (RF) and extreme gradient boosting (XGBoost) classification algorithms were used to predict retirement adjustment among participants based on their physical health, cognitive ability variables and demographic information. Regularized logistic regression, a linear perspective, was used as a reference model.

In contrast to linear models, the random forest can handle stronger non-linear relationships between the features and the target and often reaches satisfying performance with less effort and fewer computational resources (Pargent et al., 2023). XGBoost is a gradient-boosting method that optimizes parameters by iteratively explaining the model residuals. It is well-known in various research fields due to its high efficiency and accuracy (Huang et al., 2018; Sagi and Rokach, 2018).

**Figure 3:**
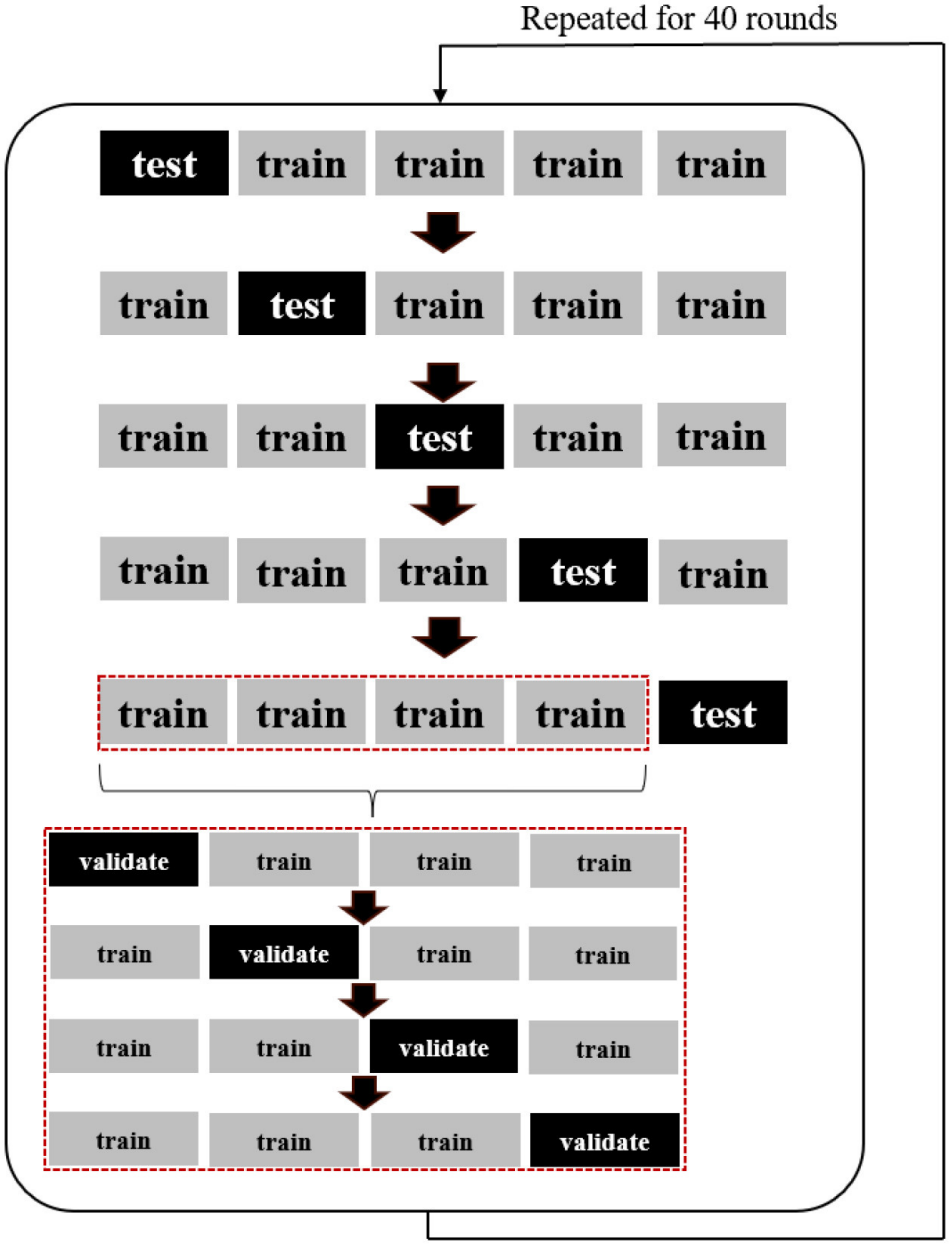
The repeated nested k-fold cross-validation framework utilized in this study.

*K*-Fold cross-validation was used to minimize the overfitting risk and enhance the model's generalizability. This was done by splitting the data into *k* number of groups; each unique group was held out as test data while the remaining *k-1* groups were used as training data (Hastie et al., 2009). This method evaluates the model's performance by dividing the data set several times to reduce the bias and variance caused by a single random partitioning of the data into the training set and the test set. In this approach, five-fold cross-validation was used for model evaluation (outer split); the data were randomly divided into five subsets; one of the five data sets was used as the test set, while the remaining four were used as the training set. four-fold cross-validation was used for nested hyperparameter optimization (inner split), and the training data were randomly split into four subsets. Three subsets were used to estimate parameters, while the other one took turns to test whether the model's hyper-parameters estimated were optimal. The cross-validation process was repeated for 40 rounds, resulting in predictions from 200 testing sets to obtain mean performance scores. The repeated nested *k*-fold cross-validation framework is illustrated in Figure 3.

Model performance was evaluated using the accuracy score, F1 score (i.e., a weighted average of the precision and sensitivity), and the area under the receiver operating characteristic curve (ROC_AUC). Finally, we used the SHapley Additive exPlanations (SHAP; Lundberg and Lee, 2017) analytic method to obtain insight into interpreting the results. SHAP values can provide both local (each prediction) and global (overall) explanations regarding the outcome of the prediction (Cooray et al., 2021). Each feature variable is allocated a SHAP value for a given prediction, signifying its importance in the prediction process. The importance of each characteristic variable in the prediction process was clarified from the SHAP value.

## 4 Results

### 4.1 Population demographics

Among 1,314 participants in the analytical sample, the imputed dataset showed that 30.29% (*n* = 398) were identified as achieving successful retirement adjustment, while 69.71% (*n* = 916) were identified as unsuccessful retirement adjustment. Table 1 displays demographic information for the two groups of participants.

**Table 1:** Descriptive statistics of sample characteristics.

**Variable**	**Successful adjustment (*****n*** **=** **398)**		**Unsuccessful adjustment (*****n*** **=** **916)**	
	***n***	**%**	***n***	**%**
**Gender**				
Male	222	55.78	222	55.78
Female	176	44.22	176	44.22
**Aere type**				
Urban	285	71.61	285	71.61
Rural	113	28.39	113	28.39
**Education**				
Literate	371	93.22	371	93.22
Illiterate	27	6.78	27	6.78
**Marital status**				
Married	368	92.46	368	92.46
Single/divorced/widowed	30	7.54	30	7.54
**Income (annual)**				
< 10,000CNY	105	26.38	105	26.38
10,000–30,000CNY	143	35.93	143	35.93
30,000–50,000CNY	90	22.61	90	22.61
50,000–100,000CNY	47	11.81	47	11.81
>100,000CNY	13	3.27	13	3.27

N = 1,314. Statistics originate from the imputed dataset.

CNY, Chinese Yuan.

### 4.2 Model evaluation and comparison

Table 2 presents the performance of all models evaluated in this study. Compared with the logistic regression of the baseline model, the machine learning model performed better. In comparison to the XGBoost model, random forest model predictions were slightly more accurate [accuracy: 80.62%, 95% CI = (75.68%, 84.16%); F1 score: 81.81%, 95% CI = (77.47%, 85.14%); AUC: 90.57, 95% CI = (86.99%, 93.72%)].

**Table 2:** Mean performance metrics for the model predictions.

**Models**	**Accuracy**		**F1 score**		**AUC_ROC**	
	***M*** **(%)**	**95% CI (%)**	***M*** **(%)**	**95% CI (%)**	***M*** **(%)**	**95% CI (%)**
LR	55.73	[50.68, 60.11]	57.61	[52.48, 62.47]	58.46	[53.45, 63.78]
RF	80.62	[75.68, 84.16]	81.81	[77.47, 85.14]	90.57	[86.99, 93.72]
XGBoost	80.35	[76.76, 84.48]	81.67	[77.81, 85.35]	89.73	[85.73, 93.29]

LR, logistic regression; AUC_ROC, area under the receiver operating characteristic curve.

### 4.3 Feature importance

**Figure 4:**
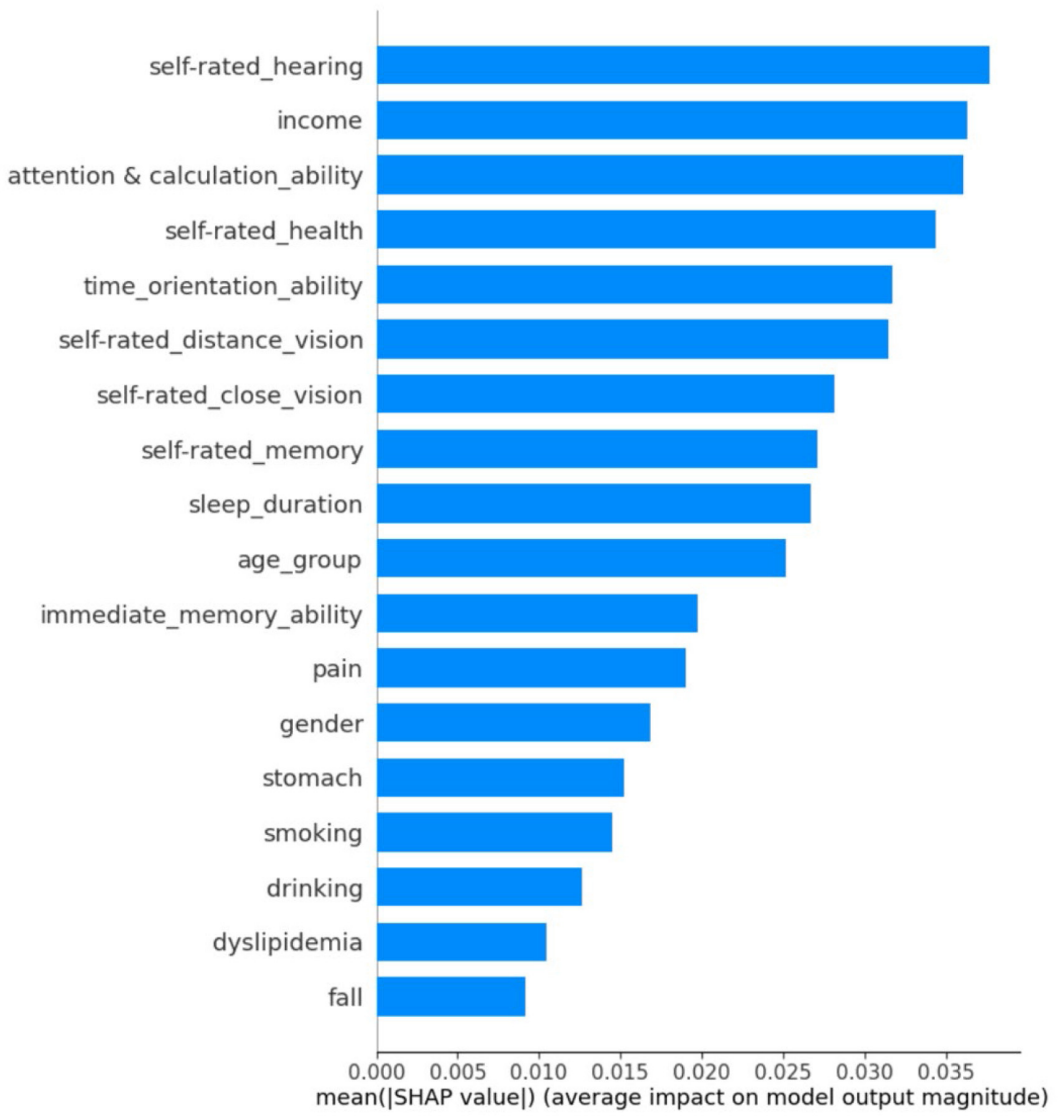
Feature importance estimated within the random forest model for predicting retirement adjustment. Feature importance was assessed using the mean of the absolute SHAP values, where greater values indicate higher importance. Information about the 18 important feature variables was displayed in the figure.

**Figure 5:**
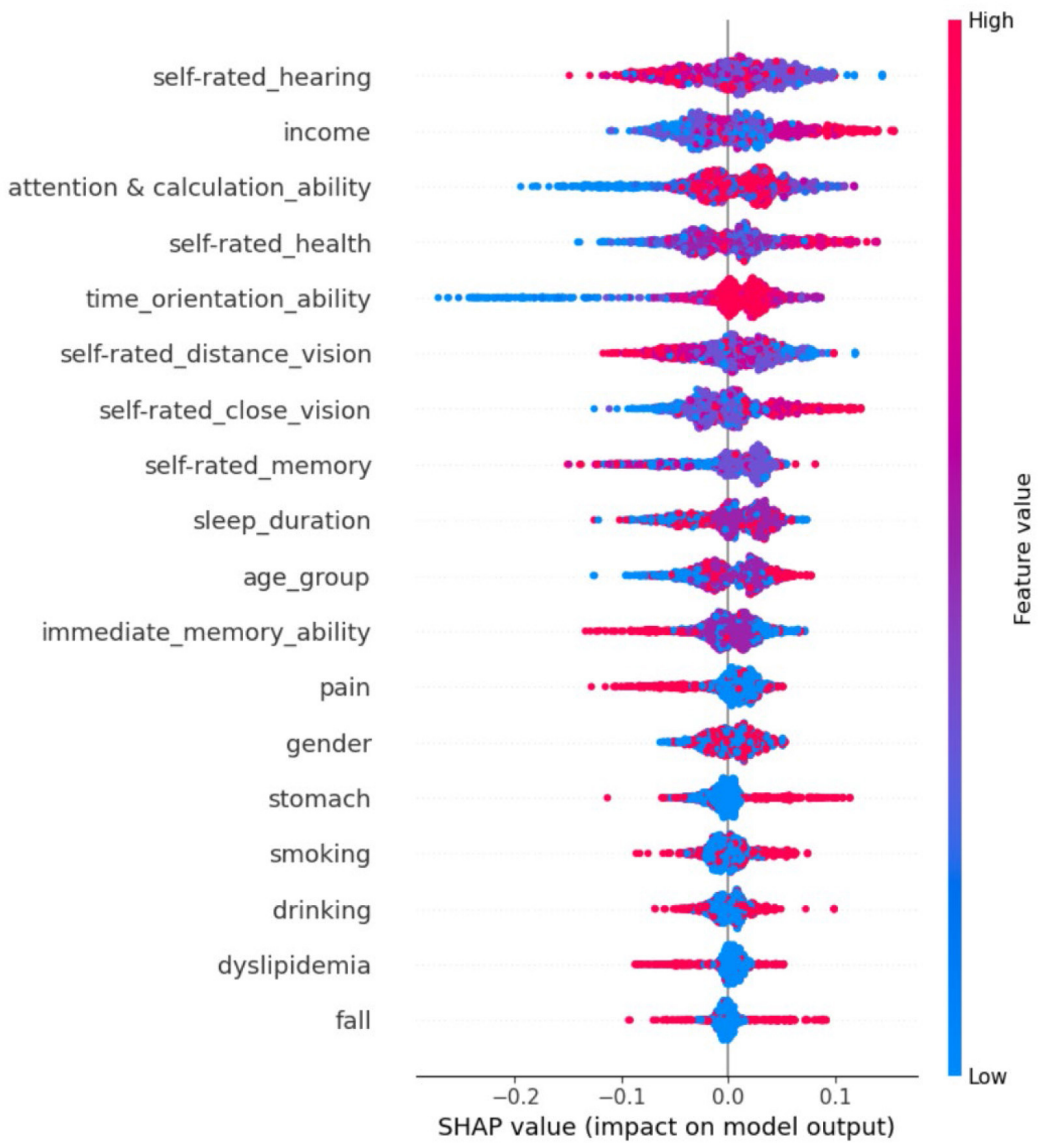
A visual explanation of the random forest prediction model. In a particular prediction, each feature was assigned a SHAP value. Positive values indicated the prediction of successful adjustment, whereas negative values indicate the prediction of unsuccessful adjustment. The encoding of feature variables is shown in Appendix B.

Figures 4 and 5 visualize the 18 important feature variables (those with the greatest |SHAP| values) within the random forest model for predicting retirement adjustment.

#### 4.3.1 Cognitive ability factors

**Figure 6:**
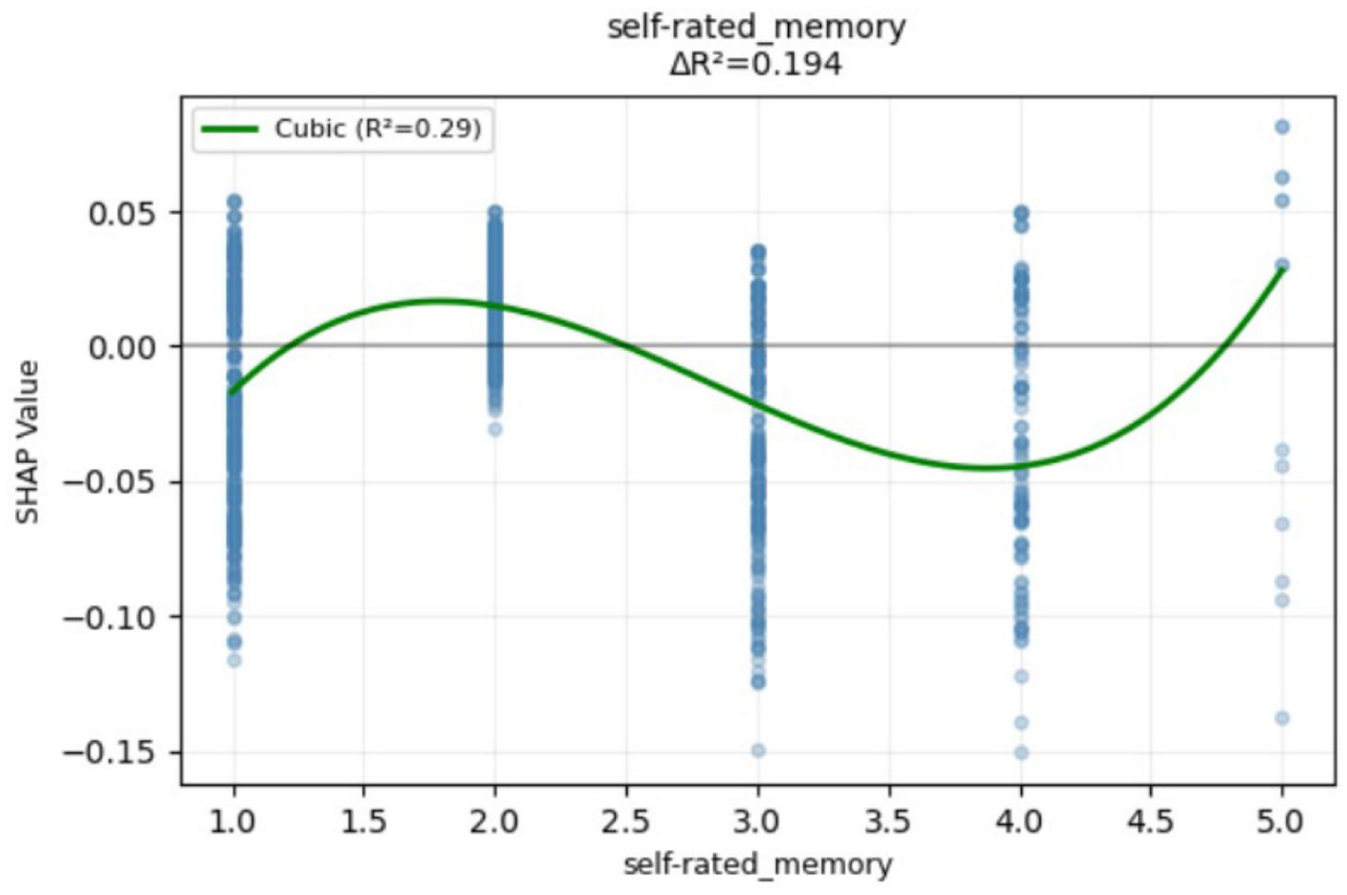
Non-linear SHAP dependence plot for self-rated memory.

Cognitive ability factors played an important role in retirement adjustment. Low attention and calculation ability and low time orientation ability were associated with unsuccessful retirement adjustment. The relationship between self-rated memory and retirement adjustment displayed a non-linear pattern, where both high self-rated memory and low self-rated memory increased the risk of unsuccessful retirement adjustment. The variation trend in non-linear relationships is detailed in Figure 6. High immediate memory ability was more strongly associated with unsuccessful retirement adjustment. Exploratory moderator analyses were conducted to investigate whether the non-linear predictive effect of self-rated memory was attributed to interactions with other predictors. The results revealed that no significant interactions were found (see Appendix C).

#### 4.3.2 Physical health factors

**Figure 7:**
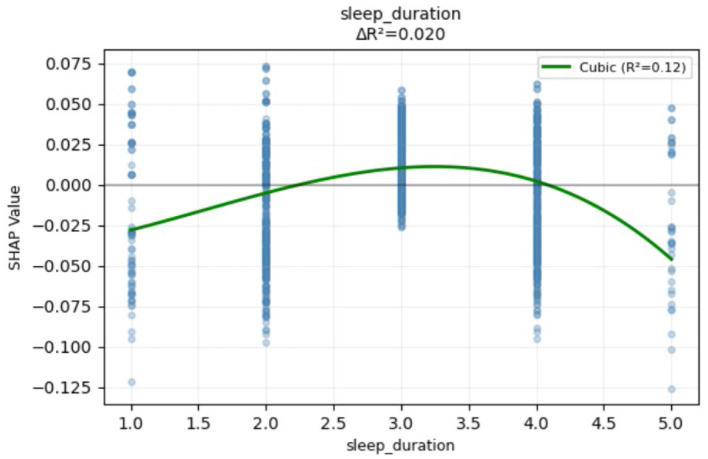
Non-linear SHAP dependence plot for sleep duration.

Self-rated health variables were another critical factor for predicting retirement adjustments. Self-rated hearing played the most important role in retirement adjustment and was negatively associated with retirement adjustment. High self-rated hearing ability was associated with unsuccessful adjustment and low self-rated hearing ability was associated with successful adjustment. High self-rated health tends toward successful retirement adjustment and low self-rated health tends toward unsuccessful retirement adjustment. Self-rated vision ability variables were also important factors in predicting retirement adjustment; high self-rated close vision ability was associated with successful retirement adjustment, but self-rated distance vision was the opposite. The third important category was objective health factors. Pain was associated with unsuccessful retirement adjustments. Stomach problems, dyslipidemia, and falls were associated with retirement adjustments status with the combination of other factors, which presents a bidirectional relationship. From the perspective of health-related lifestyle factors, sleep duration and retirement adjustment also displayed a non-linear relationship. The pattern of non-linear relationships is presented in Figure 7. Smoking and drinking, in combination with other factors, were associated with retirement adjustment status, showing a bidirectional relationship. Exploratory moderator analyses were conducted to investigate whether the non-linear predictive effect of sleep duration was attributed to interactions with other predictors. The results revealed that no significant interactions were found (see Appendix C).

#### 4.3.3 Demographic factors

Finally, from the perspective of demographic variables, income also played an important role in retirement adjustment and high income was associated with successful retirement adjustment and low income was associated with unsuccessful retirement adjustment. Individuals who retire early were more likely to unsuccessfully adjust, and there was no noticeable gender difference in retirement adjustment.

## 5 Discussion

Nowadays, retiring with good health and remaining active are largely expected life stages (Bauger and Bongaardt, 2016), and the image of retirees as being unemployed and having no social value is no longer valid (Weiss, 2005). Our study aimed to predict retirement adjustment from a physical health and cognitive ability perspective with longitudinal data from China.

Previous research on retirement adjustment was mainly based on the traditional linear perspective, which produces limited accuracy rates. Considering the potentially powerful link between multiple physical health and cognitive ability variables and retirement adjustment, this study innovatively applies machine learning techniques. Compared with the traditional linear model, this method achieves a qualitative leap in prediction accuracy because it can deal with the non-linear relationships and high-dimensional features in the data.

The results suggest that physical health and cognitive ability variables, alongside key demographic variables already established as predictors of retirement adjustment, can be used as effective indicators to predict retirement adjustment. A further difference to previous studies is that our study adopted more specific physical health and cognitive ability indicators as predictors, whereas previous research over-relied on general subjective physical health or cognitive ability indicators. Through SHAP value analysis, it was found that self-rated hearing, income, attention and calculation ability, self-rated health, and time-orientation ability were the main significant predictors of whether individuals successfully adjust to retirement. Other variables were also identified as significant predictors, such as self-rated vision, self-rated memory, sleep duration, and time orientation ability.

Based on longitudinal data, our study emphasizes the critical and dynamic impact of predictors on the changes in depression and life satisfaction, rather than the levels of depression and life satisfaction at a single point in time. The study not only extends previous research in the field of retirement adjustment, but also provides policymakers, health managers, and older adults with valuable information for promoting healthy aging.

We initially grouped 37 variables and focused on the 18 predictors selected by the random forest permutation importance selection algorithm. These features correspond to five categories, with cognitive ability factors playing a relatively important role in retirement adjustment, followed by subjective health variables, objective health factors, and health-related lifestyle factors. In addition, demographic variables, such as income and age, were also powerful in predicting retirement adjustment. The contribution of each category is discussed below.

Demographic variables were found here to be important predictors of retirement adjustment. Income was the second critical predictor and people with high income tended to successfully adjust to retirement. Topa et al. (2011) found that objective income was positively correlated with retirement adjustment. Higher income provides financial security, reduces stress related to financial instability, and allows for greater access to resources that enhance quality of life during retirement. Individuals with higher incomes can more easily afford healthcare, leisure activities, and social engagements, all of which contribute to a smoother transition and greater overall satisfaction in retirement. Wong and Earl (2009) also stated that higher income predicts better retirement adjustment. Age exhibited a non-linear relationship with retirement adjustment, people who retired early tended to unsuccessfully adjust to retirement. Previous studies found strong associations between individuals who retired early and poor mental health function (Harkonmäki et al., 2006) as well as poor physical health (De Breij et al., 2020). In contrast, people who retired at an appropriate age were more likely to successfully adjust to retirement. This might be because an appropriate age implies that people have more reasonable expectations about retirement, reducing the likelihood of psychological conflicts. However, people who retired late show a mixed pattern, which means late age for these individuals might combine with other factors to impact adjustment. It can be observed that the impact of delayed retirement may depend on the reasons for retiring. Figueira et al. (2022) identified that workers with higher autonomous motivation—driven by intrinsic factors—exhibited greater vigor and lower job stress, leading to better retirement adjustment, while those driven by external pressures experienced higher job stress and exhaustion. Similarly, a study by Jacques and Rouse (2019) found that intrinsic work motivation fosters a smoother transition into retirement, whereas external constraints may negatively affect retirement adjustment. Therefore, if the delay is due to external factors, it may negatively affect retirement adjustment. However, if the delay is driven by intrinsic motivation or self-fulfillment, it is likely to be more beneficial for retirement adjustment.

In the current study, cognitive ability was divided into time orientation ability, attention and calculation ability, immediate memory ability, and self-rated memory. These four aspects of cognitive ability contributed relatively highly to the overall ranking for the prediction of retirement adjustment, indicating the crucial importance of cognitive abilities to the prediction of retirement adjustment. Attention and calculation ability contributed the most to retirement adjustment. People with low attention and calculation ability tended to have unsuccessful retirement adjustment. This accords with findings by Sarabia-Cobo et al. (2020) showing that low attention was positively correlated with lower wellbeing in old adults after retirement. Time orientation ability and immediate memory ability also affected retirement adjustment. People who have low time orientation ability tended to have unsuccessful retirement adjustment, consistent with previous studies (Wu, 2021). People who have high immediate memory ability tended to unsuccessfully adjust to retirement, which differs from previous studies. This phenomenon can be explained by the Processing Efficiency Theory (Eysenck and Calvo, 1992). This theory suggests that individuals may attempt to compensate for decreased processing efficiency by exerting more effort. Strong immediate memory ability implies heightened sensitivity to environmental stimuli and high encoding efficiency. However, it may also lead to information overload or over-analysis. In anxiety-inducing situations, such individuals are more prone to overprocessing, which can negatively impact task performance or emotional adjustment, thereby compromising retirement adjustment. Furthermore, Sarabia-Cobo et al. (2020) found that the average scores of immediate memory and delayed memory of the retired older adults were lower than those of the occupationally active older adults, however, the relationship between immediate memory ability and retirement adjustment was not found in their study.

Moreover, in the current data the relationship between self-rated memory and retirement adjustment displayed a non-linear pattern, where both high self-rated memory and low self-rated memory increased the risk of unsuccessful retirement adjustment, and moderate self-rated memory contributed to successful retirement adjustment. In fact, with age increases, individuals' memory ability gets worse. A high or low self-rated memory score may reflect a dissociation between subjective perception and objective cognitive performance. This phenomenon may also be linked to lateral self-evaluation in social comparison theory (Festinger, 1954), as retirees often reference their peers when evaluating cognitive abilities. When such social comparisons generate either overly optimistic or pessimistic self-assessments, individuals develop biased self-perceptions that lack objectivity. A low self-rated memory can harm retirement adjustment, leading to frustration and reduced self-esteem. A high self-rated memory may create the illusion of “good memory” and prevent individuals from seeking support or adopting proactive coping strategies, leaving them unprepared for memory-related challenges. Unrealistic expectations can increase psychological pressure, strain social relationships, and hinder necessary adaptations, making the transition to retirement more difficult. Previous research has found that less pessimism, rather than more optimism, was associated with a lower risk of mild cognitive impairment and dementia. This suggests that an overly optimistic view may not necessarily protect against cognitive decline (Sachs et al., 2023).

The relationship between self-rated memory and retirement adjustment differed from previous findings on the linear negative impact of retirement on memory (Bianchini and Borella, 2016) and provided a new perspective for the study of retirement adjustment. From the above results, we found that different variables of cognitive ability had different effects on retirement adjustment. Future research needs to examine the roles of different cognitive ability indicators separately, and it is necessary to distinguish the unique roles of objective indicators and subjective indicators on retirement adjustment, as self-rated memory exhibits a non-linear relationship with retirement adjustment.

Subjective health variables were strong predictors of retirement adjustment, similar to the results of Stephan et al. (2008), who demonstrated that subjective health significantly affected retirement satisfaction. Self-rated health was positively correlated with retirement adjustment. People with high self-rated health tended to show successful retirement adjustment, which is consistent with previous studies (Hansson et al., 2019; Lowis et al., 2011).

Paradoxically, individuals who have higher self-rated hearing and distance vision ability may exhibit unsuccessful adjustment, whereas those who report higher close vision tended toward successful retirement adjustment. On the one hand, as is the case for self-rated memory, the finding reflects a dissociation between subjective perception and objective performance. Elevated self-ratings of hearing or distance vision ability may stem from peer comparisons (Festinger, 1954), thereby preventing objective self-assessment. This inhibits the adoption of protective measures and proactive coping strategies, ultimately impairing retirement adjustment. On the other hand, this seemingly counterintuitive finding can be interpreted through the lens of role theory (Ashforth, 2001), which posits that individuals derive identity and psychological wellbeing from the roles they occupy. Those with high sensory abilities are likely to have held roles that relied heavily on hearing and distance vision—such as leadership, communication, or mobility-intensive professions. Upon retirement, the loss of these roles may lead to a sense of underutilization and identity disruption, hindering psychological adjustment. In contrast, close vision is closely associated with activities common in post-retirement life, including reading or using digital devices. Individuals with higher rated close vision may thus find it easier to maintain engagement and autonomy in daily life. Furthermore, retirees who perceive themselves as still physically and cognitively capable, yet are socially or structurally excluded from contributing, may experience dissonance and dissatisfaction, further complicating their adjustment to retirement. Akuffo et al. (2021) concluded that both self-reported farsightedness and myopia were associated with a higher probability of psychological distress, which supported the predicted result of self-rated close vision ability in this study. In conclusion, all self-rated health indicators play a vital role in retirement adjustment. Notably, the roles of self-rated health and self-rated hearing and visual abilities are not entirely equivalent. Apart from self-rated close vision ability, self-rated hearing and distance vision ability may encompass more psychosocial attributes.

According to the resource-based dynamic perspective, retirement adjustment is a process involving individual resources and change. The resources in this theory are mostly objective resources, such as physical, cognitive, financial, and social resources, but we found that subjective perception is also an important kind of resource for retirement adjustment, even surpassing the importance of some objective indicators. Many studies have shown that subjective health variables are vital for retirement satisfaction (Fouquereau et al., 2005; Guerriero Austrom et al., 2003; Kim and Moen, 2002). Moreover, the relationship between subjective health and life satisfaction in retirement is consistently found across cultures (Fouquereau et al., 2005). Therefore, our results enrich the dynamic resource adjustment theory and emphasize the importance of the subjective perception of resources. Additionally, it is important to note that a higher level of subjective perception of resources does not always lead to better outcomes, especially when it is inconsistent with objective resources. Excessive or blind self-evaluation can negatively impact retirement adjustment. Excessive self-evaluation of an ability may reflect a coping mechanism in response to the threat of aging stereotypes, further impacting adjustment to retirement.

Objective health factors were also an important category in predicting retirement adjustment. People who experienced pain tended to be unsuccessful in retirement adjustments, consistent with previous studies (Zambelli et al., 2021), and not experiencing pain had no effect on retirement adjustment. Stomach problems, dyslipidemia, or falls might have different effects on retirement adjustment because of other factors, such as how retirees think about their diseases. But the absence of these diseases has no effect on retirement adjustment. Previous studies have found that objective health diseases have a different impact on an individual's retirement adjustment, but the effect depends on other factors, such as the attitude toward the disease (Tindle et al., 2010). Tindle et al. (2010) found that worsening disease can lead to unsuccessful retirement adjustments, but that the influence can be adjusted through an individual's attitude. A positive attitude can transition to successful retirement adjustment, while a negative one can lead to unsuccessful retirement adjustment.

Although our exploratory analyses did not detect significant interaction effects between subjective and objective health variables in predicting retirement adjustment, it is worth noting they might be working through interdependent mechanisms rather than operating in isolation. One prominent mechanism involves comparative appraisal processes, whereby individuals continuously evaluate and compare their internal sense of wellbeing (subjective health) against external health indicators or diagnoses (objective health). This comparison can lead to a sense of congruence or incongruence, significantly impacting overall coping strategies (Hagger and Orbell, 2003), which potentially influences retirement adjustment. Furthermore, protective cognitive mechanisms play a crucial role. Specifically, positive subjective health perceptions can act as a psychological buffer or resilience factor, mitigating the negative psychological and behavioral consequences associated with objective health limitations. This buffering effect may enhance coping efficacy, improve adherence to treatment regimens, foster greater engagement in beneficial health activities (Taylor and Brown, 1988; Calvey et al., 2024), and ultimately contribute to better retirement adjustment.

Regarding lifestyle factors, the relationship between sleep duration and retirement adjustment displayed a non-linear pattern. Too short or too long sleep duration led to unsuccessful retirement adjustment, and moderate sleep duration was conducive to successful retirement adjustment. Studies have shown that people who sleep < 4 h/night or more than 10 h/night have an increased risk of developing chronic kidney disease (Sun et al., 2021), which indicates the potential risks of too much or too little sleep, and may subsequently affect retirement adjustment. Drinking alcohol and smoking also relates to retirement adjustment with the combination of other factors, which may promote successful or exacerbate unsuccessful retirement adjustment. While refraining from drinking and smoking has no effect on retirement adjustment. Previous research has identified dual effects of alcohol consumption. The negative association between drinking and retirement adjustment has been found (Fishleder et al., 2016). Besides, van Gils et al. (2021) found that older adults drink alcohol to cope with psychological distress. From this aspect, drinking and smoking can help older adults reduce psychological distress and contribute to successful retirement adjustment. This relationship might also be driven by the context of drinking and smoking, drinking and smoking in the context of socializing may have positive effects, helping to reduce psychological distress and contributing to successful retirement adjustment, while drinking and smoking alone may have detrimental effects, worsening retirement adjustment.

In summary, the present study employed machine learning methods to predict retirement adjustment based on a diverse set of variables, including objective and subjective physical health and cognitive ability, as well as demographic and lifestyle factors. Using longitudinal data and more specific indicators of physical health and cognitive ability, the study developed a more accurate predictive model for retirement adjustment than previous studies. Notably, the results revealed that among all predictors of retirement adjustment, subjective health variables were equally important as objective variables. Furthermore, the relationships between predictors and retirement adjustment were found to be complex. These relationships could not be explained solely by simple linear trends, some exhibited linear patterns, while others followed non-linear patterns. For non-linear predictors, exploratory moderator analyses revealed that none of the non-linear predictors exhibited significant interactions with other variables in the current study. This absence of detectable interaction effects may stem from the predominance of categorical variables in our study, which can reduce sensitivity for identifying interaction effects. It may also stem from the fact that we did not detect the moderating effects of other variables that were not considered in the current study.

Additionally, many dichotomous variables demonstrated multifaceted effects on retirement adjustment, with their influence varying depending on other variables. These findings suggest that the process influencing retirement adjustment is complex, involving not only the impact of objective resources, as dynamic resource adjustment theory theorized, but also the subjective evaluations triggered by such changes. Moreover, these subjective evaluations reflect individuals' attitudes toward retirement and aging, which are equally important.

The dynamic resource adjustment theory emphasizes the vital role of resources and the changes in these resources over time. Our research highlights the importance of subjective resources and the complexity of different kinds of resources, acknowledging that different types of resources can affect individuals in varying ways. Furthermore, the current study places particular importance on the role of resources prior to retirement. For society, policymakers, and individuals, it is essential that prevention and intervention strategies are implemented before retirement, as this approach is more effective in addressing post-retirement adjustment difficulties. In addition, the Retirement Transition and Adjustment Framework focuses on the alignment between individual and environmental demands. Our research suggests that subjective factors play a crucial role, demonstrating that subjective evaluations must adapt to objective conditions in order to accurately assess environmental demands. From the perspective of the impact of specific disease, the subjective evaluation of disease may even outweigh objective factors in influencing adjustment directions. This reinforces the idea that both subjective and objective resources are interconnected in shaping retirement transitions. This study enriches theory related to retirement and holds practical implications for the early identification and improvement of retirement adjustment.

## 6 Limitations and implications

There are several potential limitations to our study. Firstly, our current study mainly considered physical health and cognitive ability factors related to retirement adjustment due to the limitation of the database. Still, other factors (such as bridge work, family numbers, social activities, etc.) also likely impact retirement adjustment, and these factors can be considered for inclusion in future studies. Secondly, according to the retirement time process model (Wang and Shi, 2014), the retirement process usually consists of three broad and continuous stages: retirement planning, retirement decision-making, and retirement transition and adjustment. Only the last stage was studied in this study, and the variables related to retirement planning and decision-making stages should be studied in future studies. Third, our study primarily focused on retirement adjustment during the transition phase, and the dynamic change refers to the changes in depression and life satisfaction. It only has two measure points and the time interval between wave n and wave n+1 is only 2 or 3 years, so the changes in depression and life satisfaction may be small and not easy to obtain, which may also be a contributing factor to the relatively small size of the successful adjustment group. Therefore, future research could focus on long-term retirement adjustment in the post-retirement phase and conduct more precise longitudinal tracking to observe changes in retirement adjustment. Moreover, the participants in the current study are from a Chinese population, and the surveyed group has relatively low economic income. The differences from existing research that focuses on developed Western countries may stem from cultural and economic factors. Future studies could consider validating the accuracy of the model of the current research in other groups.

Practically, our study suggests three actionable intervention pathways for policymakers and public health practitioners. First, integrating cognitive health screening and reinforcement into pre-retirement planning programs is essential. Given the prominence of attention and calculation ability and time orientation ability in predicting adjustment success, community health centers should offer validated cognitive training modules to recalibrate subjective cognitive assessments, for example, previous research has used meditation training to improve sustained attention among community-dwelling older adults (Ford and Nagamatsu, 2024). Second, public campaigns should combine objective sensory assessments with counseling on adaptive strategies, reducing the dissonance between subjective perception and functional reality. Third, given that income emerged as the second strongest predictor, policymakers should implement income-linked retirement transition schemes—for example, by permitting gradual workforce exit while maintaining partial earnings, especially among low-income populations.

## 7 Conclusions

Despite the limitations of our study, there are many advantages. At the theoretical level, according to resource-based dynamic retirement adjustment theory, we further confirm that physical health and cognitive ability resources are important resources affecting retirement adjustment, among which cognitive ability, subjective health, and objective health are the main groups of factors affecting retirement adjustment. More specifically, attention and calculation ability significantly impact retirement adjustment, and self-rated memory displayed a non-linear relationship in this regard. This suggests we should focus more on our cognitive ability and moderate our self-evaluation. It is also evident that specific physical health and cognitive ability variables affect retirement adjustment differently. This suggests that we must analyze specific issues concretely and adjust retirement life accordingly. In addition, we emphasize the importance of subjective resources, which are ignored by the dynamic resource retirement adjustment theory. Incorporating subjective resources into the dynamic resource retirement adjustment theory will render the theory more comprehensive. At the same time, it should also be noted that subjective resources are different from objective resources, and a moderate level of subjective resources is most beneficial for successful retirement adjustment. Therefore, our research not only provided important insights for the prevention and intervention of retirement adjustment but also enriched the content of dynamic resource adjustment theory. Notably, income was the most important predictor of retirement adjustment, which is also a key resource not to be ignored. Methodologically, this study serves as an applied example for interpreting and predicting retirement adjustment with machine learning. Machine learning is good at achieving high accuracy and capturing non-linear relationships among variables, extending beyond the limitations of previous linear studies. The model unveils complex relationships between physical and cognitive factors and retirement adjustment. In practice, this study provided a valuable reference for retirees, social workers, and policymakers concerned with retirement adjustment, emphasizing the promotion of healthy aging by adjusting subjective health evaluation and focusing on cognitive health. Meanwhile, retirees should analyze specific issues concretely and make targeted adjustments to retirement life.

## Data Availability

The datasets presented in this study can be found in online repositories. The names of the repository/repositories and accession number(s) can be found in the article/Supplementary material.
