# Supplementary material for: Physical health and cognitive ability factors in predicting retirement adjustment based on machine learning approach: results from the China Health and Retirement Longitudinal Study
Source: Front Psychol. 2025 Aug 20;16:1601723. doi: 10.3389/fpsyg.2025.1601723 (PMC12406306; doi:10.3389/fpsyg.2025.1601723)
Supplement: Supplementary file 1 [file Data_Sheet_1.zip › Appendix/Appendix.docx]

**Appendix A**


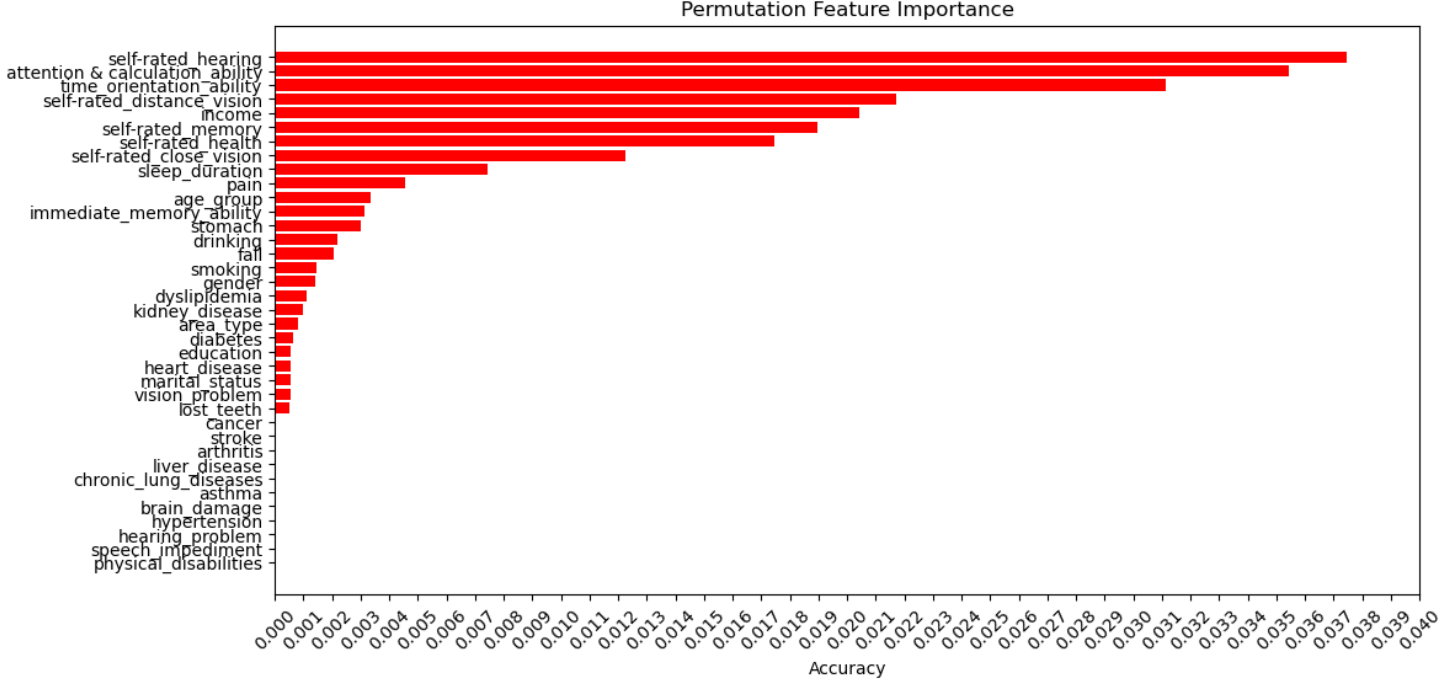


Figure A. The permutation feature importance

**Appendix B**

Table B. Predicting variables and assignments of variables

| **Variable Name** | **Encoding Type** |
| --- | --- |
| **demographic variables** |  |
| age | female: age≤50 ~ 1; 50 < age≤55 ~ 2; age > 55 ~ 3  male: age≤55 ~ 1; 55< age≤60 ~ 2; age > 60 ~ 3 |
| gender | male=1; female=0 |
| area type | urban=1; rural=0 |
| marital status | married=1; single/divorced/widowed=0 |
| education | literate=1; Illiterate=0 |
| income | <10000CNY~1; 10000-30000CNY~2;  30000-50000CNY~3; 50000-100000CNY~4; >100000CNY~5 |
| **Objective physical health** |  |
| hypertension | yes=1; no=0 |
| diabetes | yes=1; no=0 |
| cancer | yes=1; no=0 |
| chronic lung diseases | yes=1; no=0 |
| liver disease | yes=1; no=0 |
| heart disease | yes=1; no=0 |
| stroke | yes=1; no=0 |
| kidney disease | yes=1; no=0 |
| asthma | yes=1; no=0 |
| stomach | yes=1; no=0 |
| arthritis | yes=1; no=0 |
| dyslipidemia | yes=1; no=0 |
| physical disabilities | yes=1; no=0 |
| brain damage | yes=1; no=0 |
| vision problem | yes=1; no=0 |
| hearing problem | yes=1; no=0 |
| speech impediment | yes=1; no=0 |
| lost teeth | yes=1; no=0 |
| pain | yes=1; no=0 |
| fall | yes=1; no=0 |
| **Subjective physical health** |  |
| self-rated distance vision | 1=poor; 5=excellent |
| self-rated close vision | 1=poor; 5=excellent |
| self-rated hearing | 1=poor; 5=excellent |
| self-rated health | 1=poor; 5=excellent |
| **Health-related lifestyle** |  |
| smoking | yes=1; no=0 |
| drinking | yes=1; no=0 |
| sleep duration | < 4 hour~1; 4-6 hour~2; 6-8 hour~3; 8-10 hour~4;≥10hour~5 |
| **Cognitive ability** |  |
| self-rated memory | 1=poor; 5=excellent |
| time orientation ability | 1-5 points |
| attention & calculation ability | 1-5 points |
| immediate memory ability | score≤3 ~ 1; 3<score≤6 ~ 2; 6<score≤10 ~ 3 |

**Appendix C**

**
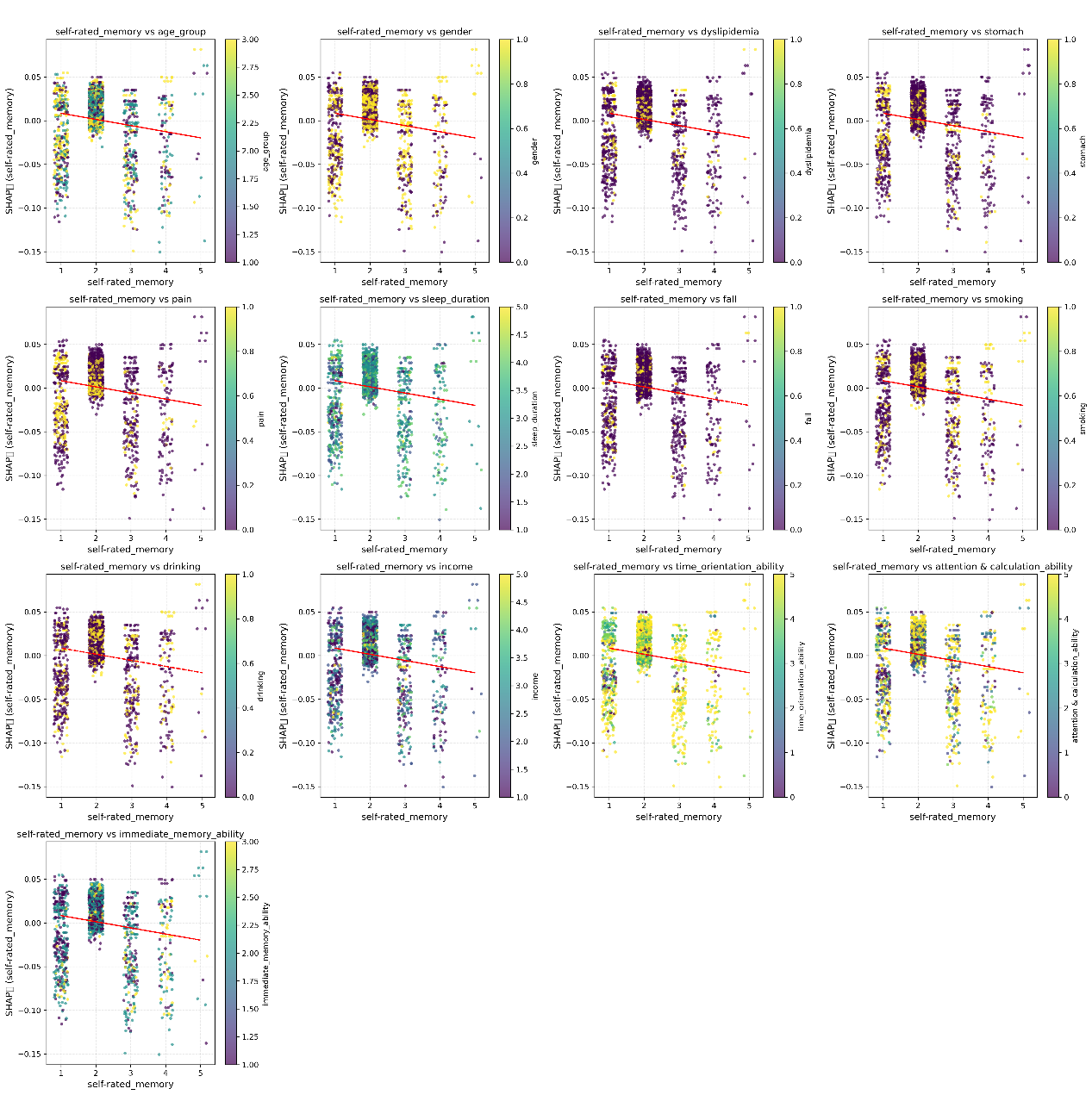
**

Figure C1. The Interaction of self-rated memory and other variables

Note. Each point corresponds to values on both axes and the color of the points indicates the values of interaction features (yellow represents high values, purple represents low values).

**
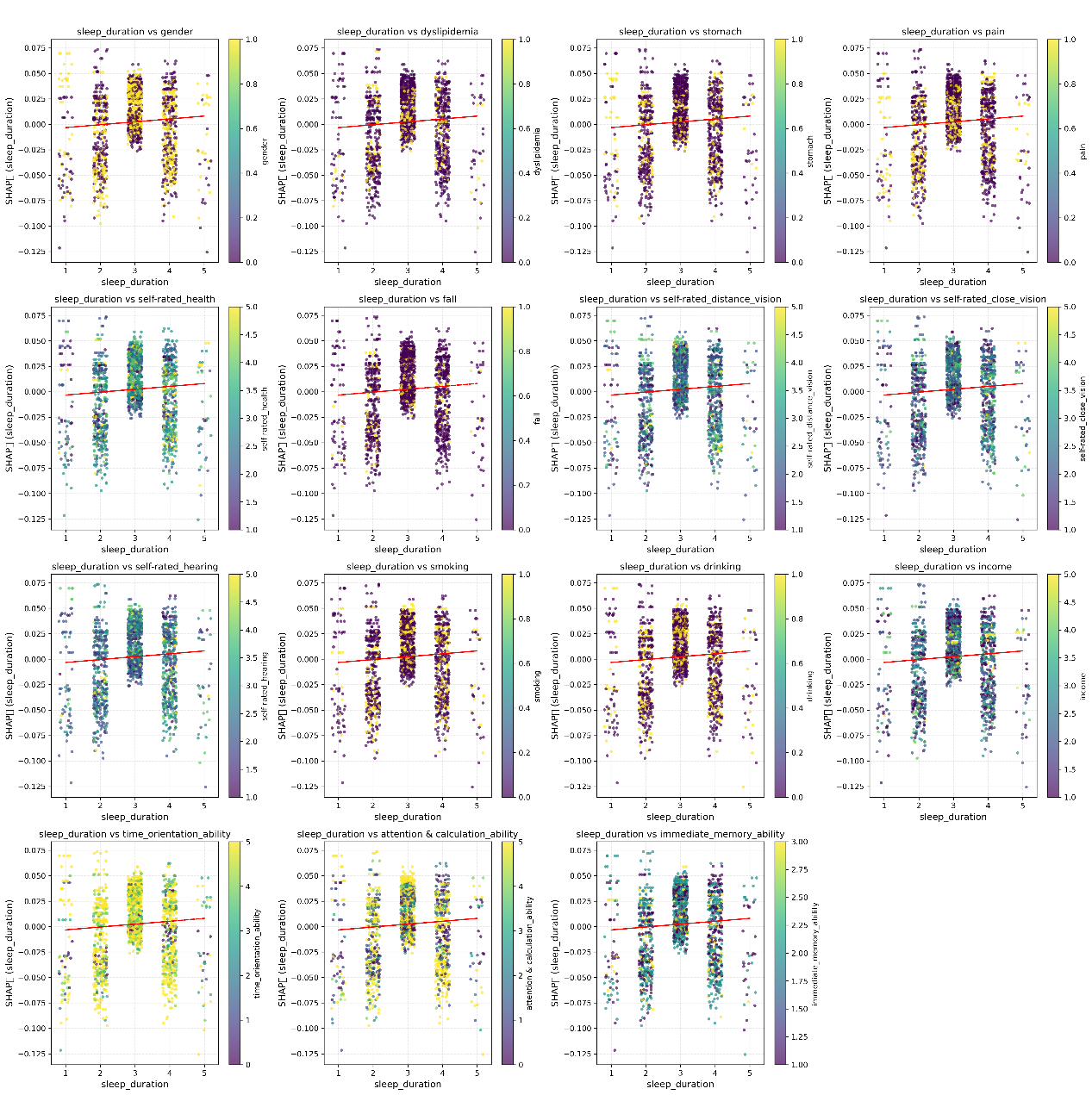
**

Figure C2. The Interaction of sleep duration and other variables

Note. Each point corresponds to values on both axes and the color of the points indicates the values of interaction features (yellow represents high values, purple represents low values).
