# Supplementary figures and images for: Physical health and cognitive ability factors in predicting retirement adjustment based on machine learning approach: results from the China Health and Retirement Longitudinal Study
Source: Front Psychol. 2025 Aug 20;16:1601723. doi: 10.3389/fpsyg.2025.1601723 (PMC12406306; doi:10.3389/fpsyg.2025.1601723)

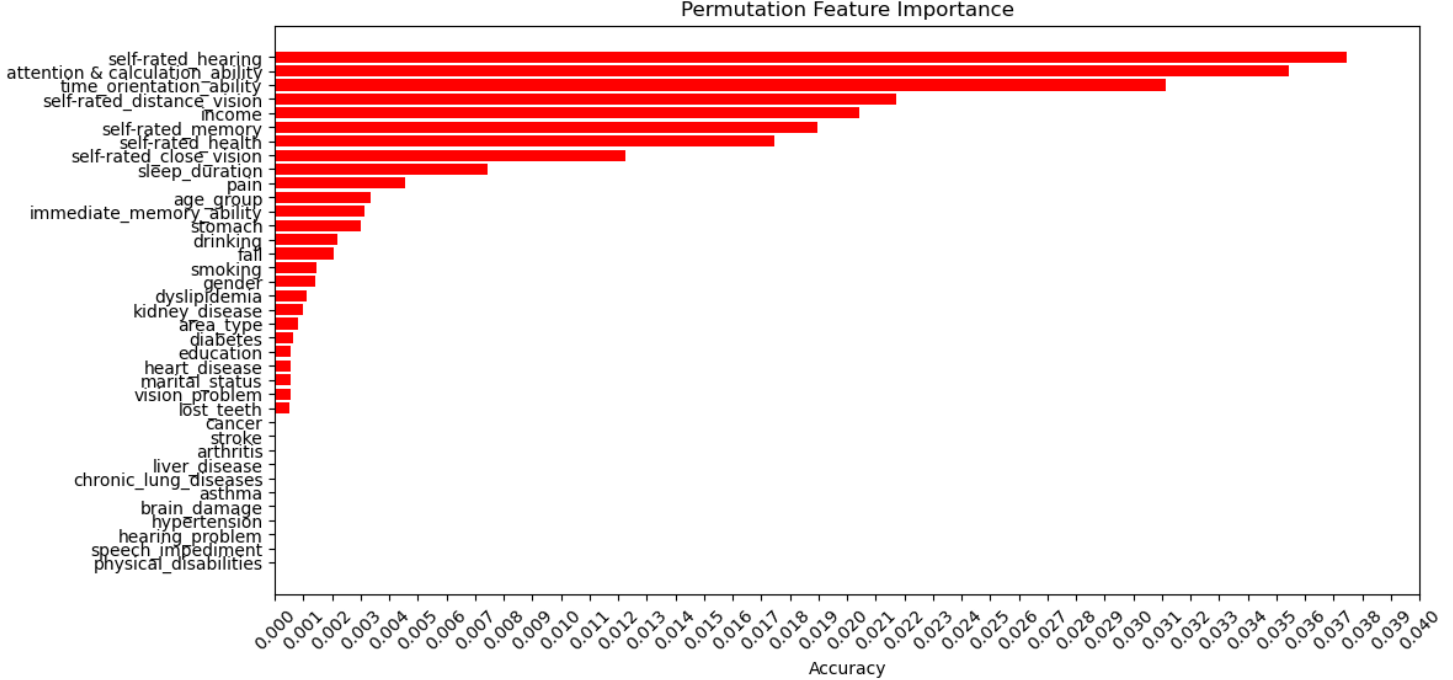

Supplement: Supplementary file 1 [file Data_Sheet_1.zip › Appendix/Appendix A figureA.png]

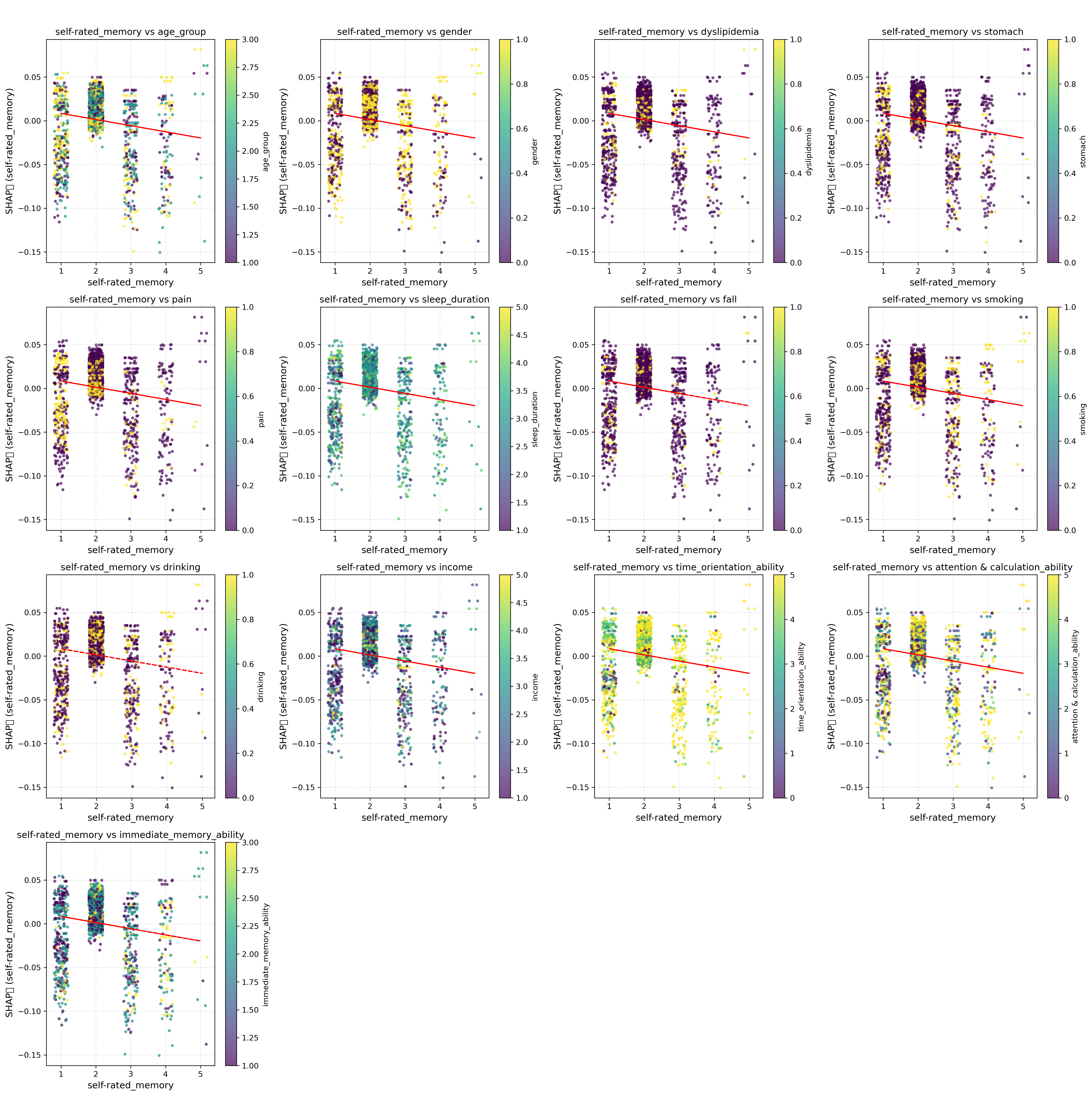

Supplement: Supplementary file 1 [file Data_Sheet_1.zip › Appendix/Appendix C figureC1.jpg]

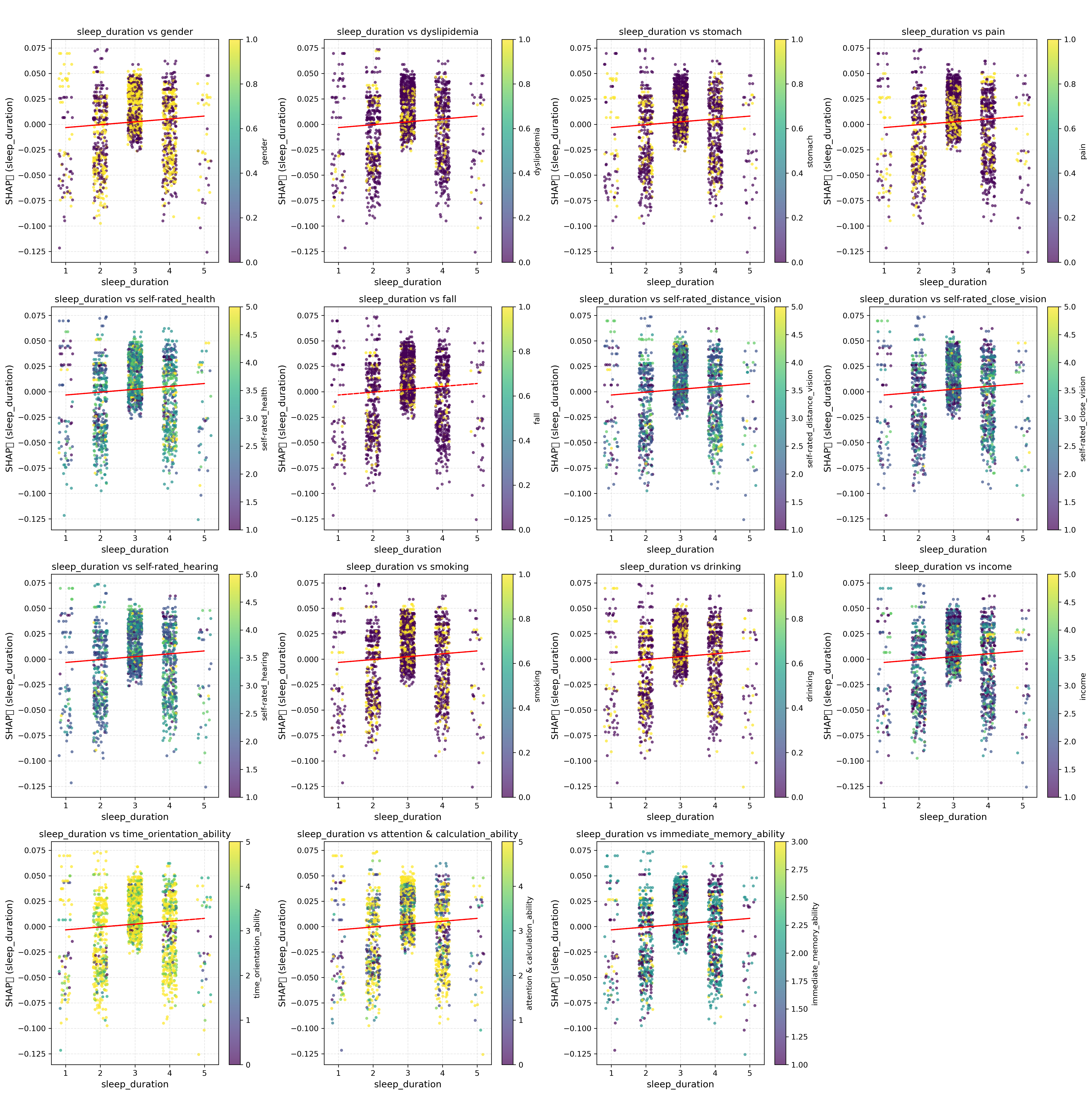

Supplement: Supplementary file 1 [file Data_Sheet_1.zip › Appendix/Appendix C figureC2.jpg]
